# Structure–Function Interactions in the Hippocampus and Prefrontal Cortex Are Associated with Episodic Memory in Healthy Aging

**DOI:** 10.1523/ENEURO.0418-23.2023

**Published:** 2024-03-26

**Authors:** Jamie Snytte, Roni Setton, Laetitia Mwilambwe-Tshilobo, M. Natasha Rajah, Signy Sheldon, Gary R. Turner, R. Nathan Spreng

**Affiliations:** ^1^Department of Psychology, McGill University, Montreal, Quebec H3A 1G1, Canada; ^2^Department of Psychology, Harvard University, Cambridge, Massachusetts 02138; ^3^Annenberg School for Communication, University of Pennsylvania, Philadelphia, Pennsylvania 19104; ^4^Department of Psychology, Princeton University, Princeton, New Jersey 08540; ^5^Department of Psychiatry, McGill University, Montreal, Quebec H3A 1A1, Canada; ^6^Department of Psychology, York University, Toronto, Ontario M3J 1P3, Canada; ^7^Montreal Neurological Institute, Department of Neurology and Neurosurgery, McGill University, Montreal, Quebec H3A 2B4, Canada; ^8^McConnell Brain Imaging Centre, McGill University, Montreal, Quebec H3A 2B4, Canada

**Keywords:** aging, compensation, episodic

## Abstract

Aging comes with declines in episodic memory. Memory decline is accompanied by structural and functional alterations within key brain regions, including the hippocampus and lateral prefrontal cortex, as well as their affiliated default and frontoparietal control networks. Most studies have examined how structural or functional differences relate to memory independently. Here we implemented a multimodal, multivariate approach to investigate how interactions between individual differences in structural integrity and functional connectivity relate to episodic memory performance in healthy aging. In a sample of younger (*N* = 111; mean age, 22.11 years) and older (*N* = 78; mean age, 67.29 years) adults, we analyzed structural MRI and multiecho resting-state fMRI data. Participants completed measures of list recall (free recall of words from a list), associative memory (cued recall of paired words), and source memory (cued recall of the trial type, or the sensory modality in which a word was presented). The findings revealed that greater structural integrity of the posterior hippocampus and middle frontal gyrus were linked with a pattern of increased within-network connectivity, which together were related to better associative and source memory in older adulthood. Critically, older adults displayed better memory performance in the context of decreased hippocampal volumes when structural differences were accompanied by functional reorganization. This functional reorganization was characterized by a pruning of connections between the hippocampus and the limbic and frontoparietal control networks. Our work provides insight into the neural mechanisms that underlie age-related compensation, revealing that the functional architecture associated with better memory performance in healthy aging is tied to the structural integrity of the hippocampus and prefrontal cortex.

## Significance Statement

Aging affects episodic memory and impacts the structure and function of the hippocampus and prefrontal cortex. In the present study, we report how functional network changes may compensate for age-related structural declines to support episodic memory. Our analyses revealed two mechanisms to maintain memory performance in older adulthood. First, greater structural integrity and enhanced within-network connectivity together were related to better episodic memory in older adults. Second, performance was maintained amid atrophy to the hippocampus when accompanied by a pattern of hypoconnectivity. Understanding how the healthy aging brain compensates for structural degeneration provides insight into disease progression, offering a unique perspective on the intersecting structural and functional trajectories that eventually converge to promote a shift from normative to non-normative aging.

## Introduction

Declines in episodic memory, our ability to encode, store, and retrieve past events (Tulving, 1972), are a hallmark of healthy aging (Salthouse, 2019). These age-related cognitive changes coincide with decreased structural integrity of the hippocampus and prefrontal cortex (PFC; Raz et al., 1997; Langnes et al., 2020). The hippocampus and PFC support episodic memory via connections to large-scale neural networks, suggesting that age-related changes to these structures may alter the functional architecture of these systems and consequently present as memory deficits.

Distinct processes can be used to retrieve episodic information, including free recall of listed items (i.e., list recall), cued recall of paired items (i.e., associative memory), and cued recall of stimulus-related information, such as the type of trial the item was presented in (i.e., source memory; Rey, 1941; Wechsler, 1945; Tulving, 1985). Age-related deficits are apparent across processes due to a deficit in relational binding—the association of items to a list, to other items, or to a particular context (McIntyre and Craik, 1987; Naveh-Benjamin, 2000). Associative and source memory tasks require participants to recall the links between items and other items, or between items and related features, necessitating this binding process (Eichenbaum and Bunsey, 1995; Chalfonte and Johnson, 1996). In contrast, while free recall is facilitated by recollecting the list and the temporal order in which items were presented, contextual information is not necessarily required for correct retrieval (Kahana, 1996; Ranganath, 2022). Critically, associative and source memory tasks provide a partial cue during the retrieval phase, which may serve as a scaffold for performance in individuals with maintained brain structure and connectivity (Josefsson et al., 2012; Stern et al., 2023).

The hippocampal and prefrontal subregions are impacted in healthy aging but exhibit somewhat distinct trajectories. The posterior hippocampus (postHC) displays reduced volume around the fifth decade of life, whereas alterations to the anterior hippocampus (antHC) are apparent a decade later (Langnes et al., 2020). Within the PFC, cortical thickness of the middle frontal gyrus (MFG) declines across the adult lifespan, with stronger impact on the caudal compared with the rostral subregion (cMFG and rMFG, respectively; Salat et al., 2004; Thambisetty et al., 2010; Dominguez et al., 2021). While volumes of the hippocampal subregions are related to source memory performance in young adults (Rajah et al., 2010; Poppenk and Moscovitch, 2011), the relationships between specific brain structures and dimensions of episodic memory may shift with age. Cortical thickness of prefrontal regions, such as the MFG, correlates with source and associative memory in older adults, potentially due to the contribution of this region to executive function (Burzynska et al., 2012; Becker et al., 2015; Guardia et al., 2023).

Alterations in connectivity may further accentuate structure–behavior relationships, or may instead play a compensatory role. The hippocampus and MFG play unique roles in episodic memory but interact with one another, each forming part of a large-scale neurocognitive system across the brain; namely, the default and frontoparietal control networks, respectively (DN and FPN; Buckner et al., 2008; Vincent et al., 2008; Yeo et al., 2011). Older adults display enhanced network integration, expressed as increased coupling between the default and frontoparietal control systems (Turner and Spreng, 2015). Resting-state functional connectivity (RSFC) within the hippocampus decreases with age (Wang et al., 2010; Panitz et al., 2021; Setton et al., 2022a,b ), whereas connectivity between the hippocampus and MFG during episodic encoding and retrieval increases with age and is related to poorer performance (Dennis et al., 2008; Ankudowich et al., 2019).

While previous studies have inspected individual pieces of this puzzle, surprisingly little work has investigated how interactions between these functional and structural individual differences impact memory (Rajah et al., 2011; Fjell et al., 2016, 2017; Snytte et al., 2022). A fundamental step toward understanding aging requires bridging this multimodal gap to reveal the neural underpinnings of age-related differences in episodic memory. Peering through this multimodal lens may help resolve differing reports on age differences in connectivity and shed light on how functional reorganization can preserve memory function.

In the present study, we investigated age differences in the relationship between brain structure, RSFC, and dimensions of episodic memory. First, we assessed how the structural integrity of the hippocampus and MFG were associated with memory performance. We predicted that region-specific relationships may differ between age groups, with the MFG playing a more important role in older adults. Next, we examined how connectivity and structural integrity of the hippocampus and MFG related to episodic memory processes. We predicted that more intact hippocampal and MFG structure would be associated with greater within-system connectivity and enhanced memory performance. Further, we predicted that functional reorganization may salvage episodic memory amid atrophy to these key structures.

## Materials and Methods

**Figure 1.**
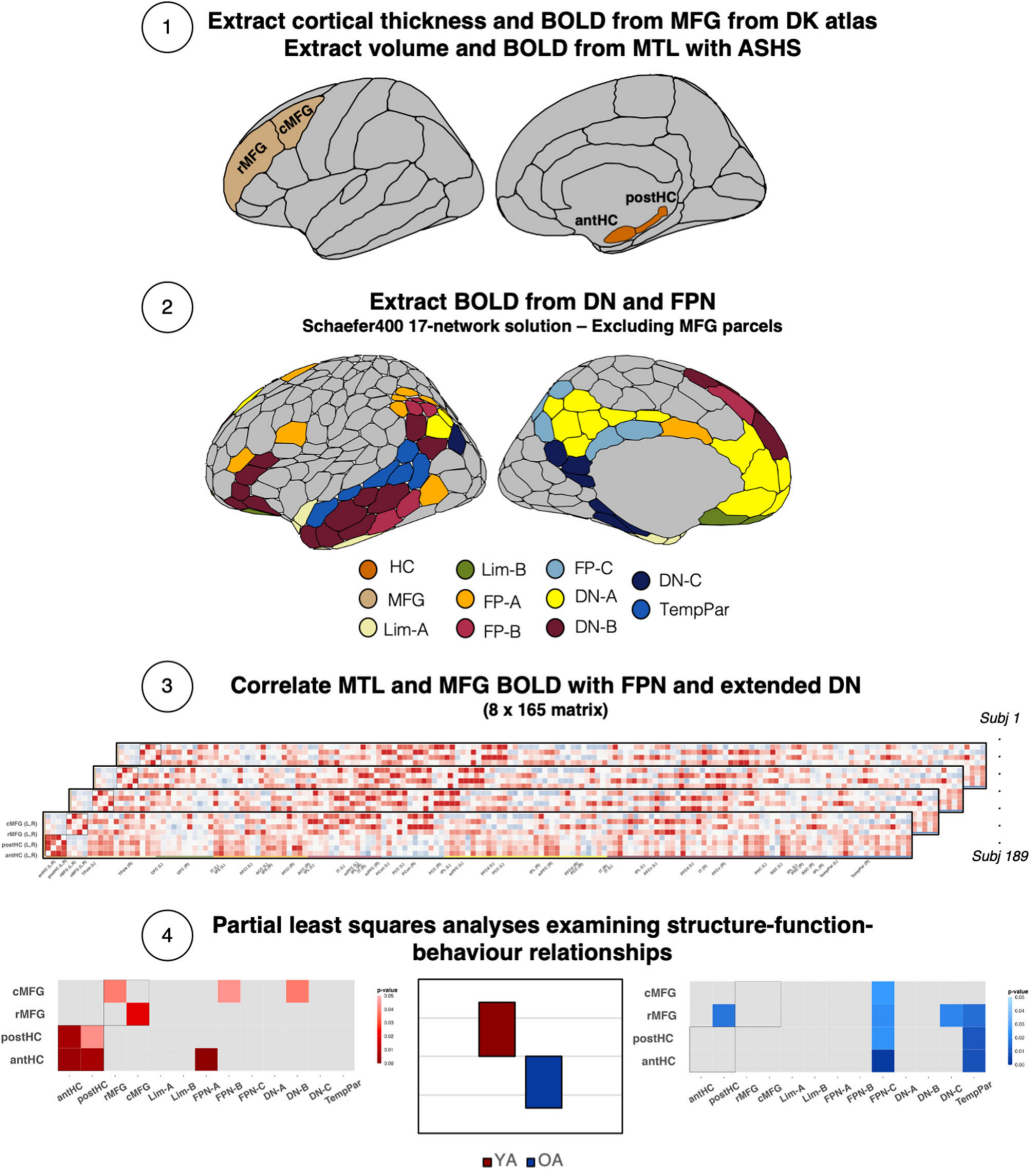
Diagram of analytic pipeline for image processing and analyses. ***1***, Cortical thickness and BOLD signal were extracted from the caudal and rostral middle frontal gyrus (cMFG and rMFG, respectively) with FreeSurfer. Anterior and posterior hippocampal volumes (antHC and postHC, respectively) and BOLD signal were extracted with ASHS. ***2***, BOLD was extracted from frontoparietal (FP) and extended default networks (DN), excluding parcels overlapping with the MFG. ***3***, Hippocampal and MFG BOLD signal were correlated with FPN-DN BOLD to create 8 × 165 matrices for each participant. ***4***, Partial least squares analyses were used to assess relationships between brain structure, memory performance, and functional connectivity.

In a deep-phenotyped sample, we applied a multimodal approach to investigate how the hippocampus and MFG support episodic memory in healthy aging (Spreng et al., 2022). We used well-established neuropsychological and laboratory-based tasks to assess three dimensions of episodic memory: item, associative, and source memory. To assess structure–function interactions, we acquired standard T1 structural MRIs to compute antHC and postHC volume and rMFG and cMFG cortical thickness (Fischl et al., 2002; Yushkevich et al., 2015; Xie et al., 2016). We collected two 10 min multiecho (ME) resting-state fMRI scans and used participant-specific masks to extract connectivity of the hippocampal and MFG subregions with neurocognitive networks of interest. This included the limbic A and B networks (LIM-A, LIM-B), the frontoparietal control subnetworks (FPN-A, FPN-B, FPN-C), the default subnetworks (DN-A, DN-B, DN-C), and the temporoparietal network (TEMP-PAR). Innovations in acquisition (ME sequences) and image processing (individualized parcellations) optimized our ability to identify how the structure and connectivity of the hippocampus and MFG support memory processing in healthy aging (Kundu et al., 2012, 2013; Chong et al., 2017). Our analyses proceeded in the following steps, also shown in Figure 1. We first examined age differences in memory performance (item, associative, and source memory) and brain structure (hippocampal gray matter volume and MFG cortical thickness) and tested for age differences in structure–behavior relationships. We next investigated age differences in functional connectivity and specifically examined how patterns of RSFC related to memory performance in the context of structural differences of the hippocampus and MFG.

### Participants

Participants in the current study included 111 young adults (mean age, 22.11 years; standard deviation, 3.22, 58.55% female) and 78 older adults (mean age, 67.30; standard deviation, 5.65, 56.41% female), resulting in a total sample size of 189 participants (Table 1). This cohort is a subsample of participants from a single site, who completed list recall, associative, and source memory tasks, and underwent two 10 min ME functional MRI (ME-fMRI) scans, as well as one standard anatomical T1-MPRAGE scan (Spreng et al., 2022). All participants were healthy right-handed adults with normal to corrected vision, and without underlying neurological or psychiatric conditions; tested and scanned in Ithaca, New York; and scored 27/30 or above on the Mini Mental State Examination (Folstein et al., 1975). Please see Spreng et al. (2022) for more information related to the participants.

**Table 1.** Demographics and age differences in memory performance, hippocampal volumes, and MFG thickness

	Young adults (*N* = 111)	Older adults (*N* = 78)
Age	22.11 ± 3.22	67.29 ± 5.65
Education	15.09 ± 1.89	17.55 ± 3.00
List recall (%)	0.72 ± 0.20	0.45 ± 0.23
Associative (%)	0.77 ± 0.14	0.49 ± 0.21
Source (%)	0.81 ± 0.13	0.68 ± 0.13
antHC volume (mm^3^)	3,675.00 ± 499.28	3,585.96 ± 444.05
postHC volume (mm^3^)	3,594.90 ± 353.05	3,414.74 ± 339.77
Rostral MFG thickness (mm)	4.94 ± 0.17	4.60 ± 0.19
Caudal MFG thickness (mm)	5.37 ± 0.19	5.01 ± 0.21

### Episodic memory measures

In the current study, the delayed recall component of the Rey Auditory Verbal Learning Task (RAVLT) was used as a measure of list recall (Schmidt, 1996). In this task from the NIH cognition toolbox, participants learned a list of 15 unrelated items and then recalled them after a 20 min delay (Denboer et al., 2014). The Associative Recall Paradigm was used as a measure of associative memory (Brainerd et al., 2013). In this task, participants learned four lists of 30 pairs of words and were then cued with single items to provide the associated word after a brief delay. Source memory was extracted from a Remember–Know task, where participants were asked to learn individual words that were presented either visually or orally (Tulving, 1985; Gardiner, 1988). At retrieval, participants completed 24 trials where they were shown previously seen or heard words (old) and 24 trials where they were shown new words. Participants had to identify whether they remembered seeing the word, an indication of recollection, or if they simply knew that they saw it, an indication of familiarity. Participants were also asked to recall source information for each word—whether it was presented visually or orally. Our measure of source memory was calculated as the total number of trials where participants correctly identified an item as old (either “remember” or “know”) and correctly identified the trial type, divided by the total number of old trials completed. We note that while this measure may be tapping source memory (i.e., linking the item with external information such as trial type), the test also may be completed correctly by recalling information intrinsic to the item such as the modality in which it was presented. For example, at the recognition phase, participants may correctly recall that “bagel” was presented during an auditory trial (retrieving the source), or they may recall the sound of the word (retrieving the item features). Thus, this measure does not purely assess source memory and must be interpreted as also potentially tapping memory for intrinsic properties of the item. Still, this task would require relational processing, tying some type of feature (whether internal or external) to the presented item. Additionally, the associative and source memory tasks both provide partial cues at retrieval; the paired word is shown in the associative memory task, and the word is shown in the source memory task.

### Neuroimaging

#### Acquisition

All images were acquired on a 3 T GE750 Discovery series MRI scanner, using a 32-channel head coil. Anatomical T1-MPRAGE scans were acquired with a T1-weighted volumetric magnetization prepared rapid gradient echo sequence (TR, 2,530 ms; TE, 3.4 ms; 7° flip angle; 1 mm isotropic voxels; 176 slices; 5 min25 s) with 2× acceleration with sensitivity encoding. Participants completed two resting-state fMRI scans (10 min06 s each) where they were instructed to remain awake, breath normally, and lie still with their eyes open in the scanner bay. These images were acquired with an ME-EPI sequence [TR, 3,000 ms; TE1, 13.7 ms; TE2, 30 ms; TE3, 47 ms; 83° flip angle; matrix size, 72 × 72; field of view (FOV), 210 mm; 46 axial slices; 3 mm isotropic voxels; 204 volumes; 2.5× acceleration with sensitivity encoding].

#### Image processing

##### Structural data

Anatomical images were preprocessed in FSL with the brain extraction tool to skull strip the brain (Smith, 2002). The medial temporal lobes were submitted to the Automatic Segmentation of Hippocampal Subfields pipeline (ASHS; Yushkevich et al., 2015). ASHS uses multiatlas label fusion to delineate regions of interest (ROIs) in individual participants, according to the submitted atlas, here the ASHS-PMC-T1 atlas (Xie et al., 2016). We extracted the anterior (head) and posterior (body and tail) portions of the hippocampus bilaterally. Segmentations were examined by two raters for gross errors.

T1 images were also submitted to FreeSurfer 6.0.1 after preprocessing for cortical reconstruction (Fischl et al., 2002). Cortical thickness measures for the caudal and rostral middle frontal ROIs were extracted bilaterally. Additionally, the measure of total intracranial volume was calculated from FreeSurfer as the sum of total gray and white matter volumes and cerebrospinal fluid and was residualized from all volumes and thickness measures, along with sex and education. Left and right measures of volumes and thickness were summed for each ROI and are consistent with previous estimates (Salat et al., 2004; Fjell and Walhovd, 2010; Langnes et al., 2020).

##### Functional data

ME-fMRI brain images were submitted to ME Independent Components Analysis (ME-ICA). ME-ICA determines the T_2_* in every voxel using the TE dependence model of BOLD signal and distinguishes BOLD signal and non-BOLD TE-independent noise (Kundu et al., 2012, 2013). This process boosts the BOLD signal-to-noise ratio, particularly in the anterior frontal and temporal regions such as the orbitofrontal cortex, temporal pole, and hippocampus, which are liable to signal dropout. Denoised BOLD component coefficient sets from ME-ICA were then mapped to a common cortical surface (fsaverage5) in FreeSurfer and concatenated. To improve the homogeneity of BOLD signal within parcels, better demarcate functional regions, and enhance our ability to detect associations between connectivity and memory performance, we applied Group Prior Individual Parcellation (GPIP; Chong et al., 2017). This step involves the generation of individualized participant-specific parcellations, where parcel labels are preserved across participants, but boundaries may shift based on an individual's parcel boundaries.

BOLD data were extracted from each of our networks of interest, which here included the limbic A and B networks (LIM-A, LIM-B), the frontoparietal control subnetworks (FPN-A, FPN-B, FPN-C), the default subnetworks (DN-A, DN-B, DN-C), and the temporoparietal network (TEMP-PAR) from the Yeo-17 network solution (Yeo et al., 2011). To extract BOLD signal from the hippocampus, ROIs from ASHS were first binarized and resampled to native functional space. BOLD data were then extracted from each of four ROIs (left and right antHC and postHC). The same procedure was applied to extract BOLD signal from the MFG, using the MFG masks from FreeSurfer (left and right rMFG and cMFG). Parcels from the Schaefer 400 atlas that overlapped with the MFG were excluded from their respective networks when creating each participants connectivity matrix. Specifically, parcels contained in the left rMFG that overlapped with our networks of interest included DN-A dorsal PFC, DN-B dorsal PFC, FPN-A lateral PFC, FPN-A ventrolateral PFC, and three FPN-B ventrolateral PFC parcels. Parcels contained in the right rMFG that overlapped with our networks of interest included DN-A dorsal PFC and medial PFC, two FPN-A lateral PFC parcels, and four FPN-B ventrolateral PFC parcels. Parcels contained in the left cMFG that overlapped with our networks of interest included two DN-B lateral PFC parcels, FPN-A lateral PFC, and FPN-B dorsal PFC. Parcels contained in the right cMFG that overlapped with our networks of interest included FPN-A lateral PFC and three FPN-B dorsolateral PFC parcels.

Functional connectivity matrices were created by computing product-moment (*r*) correlation coefficients between each pair of regions, followed by a Fisher's *r*- to *z*-transformation to normalize the correlation values and account for the varying number of BOLD coefficients for each participant. We created 8 × 165 rectangular matrices for each participant. The square portion of the matrix included four rows and columns with the hippocampus (left and right antHC, postHC) and four rows and columns corresponding to parcels within the anatomically defined rostral and caudal MFG from FreeSurfer (left and right caudal and rostral MFG)—this included parcels within the FPN and DN noted above and parcels from the Salience network-B (three lateral PFC parcels for the left and right rMFG, respectively) according to the Schaefer 400 parcel atlas (Schaefer et al., 2018). The remaining columns, creating the rectangular part of the matrix, included the FP and extended DN subnetworks. See Figure 1 and Table 2 for the regions listed in this matrix.

**Table 2.** Regions and networks of interest

Subnetwork	Regions
antHC	Left and right anterior hippocampus (antHC)
postHC	Left and right posterior hippocampus (postHC)
Limbic network A (Lim-A)	Left and right temporal pole (TP)
Limbic network B (Lim-B)	Left and right orbitofrontal cortex (OFC)
Frontoparietal network A (FPN-A)	Left and right inferior temporal gyrus (IT), intraparietal sulcus (IPS), lateral prefrontal cortex (PFCl), and anterior cingulate cortex (ACC)
Frontoparietal network B (FPN-B)	Left and right Inferior parietal lobule (IPL), inferior temporal gyrus (IT), and medial prefrontal cortex (mPFC)
Frontoparietal network C (FPN-C)	Left and right precuneus (PCun) and posterior cingulate cortex (PCC)
Default network A (DN-A; core regions)	Left and right inferior parietal lobule (IPL), dorsal prefrontal cortex (dPFC), medial prefrontal cortex (mPFC), precuneus and posterior cingulate cortex (PCun/PCC), and right inferior temporal cortex (IT)
Default network B (DN-B; dorsomedial subnetwork)	Left and right lateral temporal cortex (lT), inferior parietal lobule (IPL), dorsal medial prefrontal cortex (dmPFC), lateral prefrontal cortex (lPFC), and ventral prefrontal cortex (vPFC)
Default network C (DN-C; medial temporal subnetwork)	Left and right inferior parietal lobule (IPL), retrosplenial cortex (RSC), and parahippocampal cortex (PHC)
Temporoparietal network (TEMP-PAR)	Left and right temporoparietal cortex

### Statistical analyses

#### Age differences in episodic memory, hippocampal volume, and MFG thickness

To examine age differences in episodic memory, we compared young and older adults on measures of list recall, associative, and source memory. We computed ANCOVAs for each measure, including sex and education as covariates. Next, to assess age differences in brain structure, we compared the cortical thickness for the rMFG and cMFG and volume of the antHC and postHC between young and older adults. We computed ANCOVAs covarying for sex, education, and total intracranial volume. To examine how measures of brain structure supported episodic memory, we computed linear regression models to predict performance on each episodic memory task with our structural measures for each region (volume or thickness). We also included age group as an interaction term in each model to assess if these structure–behavior relationships differed between young and older adults. Thus, we computed 12 linear models, with memory (3) and structural measures (4) differing in each model, with the format shown below as an example. To correct for computation of multiple models, we used Bonferroni’s correction for the *p* values of the model, setting our significance threshold to *α*_Bonferroni’s_ = 0.004 (0.05/12):

Associativememory∼caudalMFGthickness+agegroup+caudalMFGthickness*agegroup.

#### Age differences in connectivity, associations with performance, and brain structure

To examine associations between brain structure, memory performance, and connectivity, we applied a behavioral-partial least squares analysis (B-PLS). PLS is a multivariate analysis that extracts latent patterns of connectivity, maximally associated here with age group, memory performance, and structural metrics (McIntosh and Lobaugh, 2004). This type of analysis was selected as it allows us to include functional, structural, and behavioral features in a single model. While univariate approaches may provide insight into which specific brain regions uniquely contribute to differences in memory performance, multivariate analyses help reveal how connectivity between multiple regions, parcels, or networks (i.e., a pattern of connectivity) together is associated with episodic memory. Given that little research has examined the multimodal relationships investigated here, a multivariate data-driven approach is well suited to resolve these high-dimensional interactions. Finally, multivariate methods enhance our ability to detect brain–behavior associations (Genon et al., 2022; Spisak et al., 2023).

The above 8 × 165 matrix for each participant was submitted to the PLS analysis, which extracts latent variables (LVs) using singular vector decomposition. Each LV consists of a singular value, indicating the amount of cross-block covariance explained by the LV and a correlation profile reflecting how the behavioral variables correlated with the latent pattern of connectivity. Permutation tests were used to test the significance of each LV (*p* < 0.05; 1,000 permutations). Bootstrapping was used to assess the stability of each edge's contribution to the connectivity profile, at a bootstrap ratio of ±1.96 (corresponding to *p* < 0.05; 500 permutations). Confidence intervals were plotted across the brain–behavior correlation profiles to assess significance of behavioral correlations with connectivity profiles; error bars that did not cross the zero on the *x*-axis indicated significant correlations (95% confidence intervals). To assess the contribution of chronological age in the PLS analyses, we examined correlations between the brain scores (how much each participant expressed the latent connectivity pattern) with chronological age. All analyses were computed in MATLAB version 9.8.0 (R2020a; MathWorks).

#### Network contributions

To understand how network-level connectivity was related to memory performance and brain structure, we examined contributions of each network of interest to the latent patterns of connectivity extracted from the PLS analyses. We constructed positive and negative adjacency matrices from each PLS pattern and assessed contributions at the network level as follows. First, we assigned each parcel to either their network assignment, according to the Yeo-17 network solution, or a hippocampal or MFG network. Next, we created a 4 × 13 matrix, by computing the average of all connection weights in a given network and calculating within- and between-network connectivity (antHC, postHC, rMFG, cMFG × Lim-A, Lim-B, FPN-A, FPN-B, FPN-C, DN-A, DN-B, DN-C, TEMP-PAR). Finally, we used permutation testing to test for significant network contributions. Network labels were shuffled for 10,000 permutations, to recompute mean within- and between-network connectivity values. Network connections significantly contributed to this pattern when the proportion of times the value of the sampling distribution was greater or equal to the empirical null distribution did not exceed 0.05.

### Code and data accessibility

All fMRI data, anatomical images, and scores on cognitive tests are available on OpenNeuro (https://openneuro.org/datasets/ds003592). Code is open access and available on OSF (http://osf.io/yhzxe/).

## Results

### Age differences in episodic memory, hippocampal and MFG structure, and structure–behavior relationships

**Figure 2.**
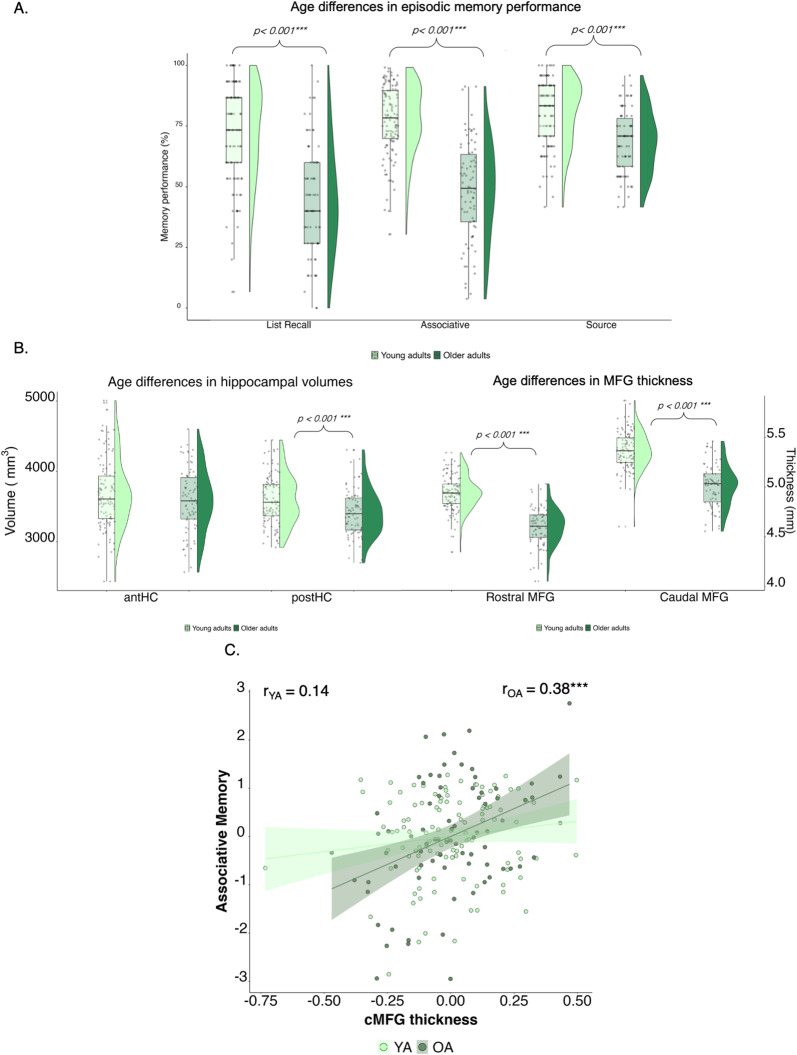
***A***, Age differences in memory performance: younger adults display better performance across list recall, associative, and source memory tasks. ***B***, Age differences in brain structure: older adults display smaller postHC volume and less rMFG and cMFG thickness. ***C***, Greater cMFG thickness predicted greater associative memory performance in older but not younger adults.

We compared young and older adults on measures of list recall, associative, and source memory to assess age differences in episodic memory (Table 1, Fig. 2*A*). We observed better performance in younger compared with that in older adults across all three mnemonic domains (list recall: *F*_(1,185)_ = 61.59, *p* < 0.001, *ω*^2^ = 0.23; associative: *F*_(1,185)_ = 110.01, *p* < 0.001, *ω*^2^ = 0.36; source: *F*_(1,185)_ = 22.84, *p* < 0.001, *ω*^2^ = 0.10). Next, we examined group differences in cortical thickness for the rMFG and cMFG and volume of the antHC and postHC. Older adults displayed lower cortical thickness within both MFG subregions (rostral MFG: *F*_(1,184)_ = 121.01, *p* < 0.001, *ω*^2^ = 0.39; caudal MFG: *F*_(1,184)_ = 117.03, *p* < 0.001, *ω*^2^ = 0.38). Within the hippocampus, older adults displayed less gray matter volume in the postHC (*F*_(1,184)_ = 14.45; *p* < 0.001; *ω*^2^ = 0.055). Age differences for the antHC were not observed (*F*_(1,184)_ = 1.90; *p* = 0.17; *ω*^2^ = 0.004).

To examine how brain structure was associated with memory performance in young and older adults, we computed linear regression analyses for each ROI and memory type, including age group as a moderator and controlling for sex, education, and total intracranial volume. We found that associative memory performance was significantly predicted by cMFG thickness and age group (*F*_(3,185)_ = 6.14; *p* < 0.001; $R_{\mathrm{adjusted}}^{2}=7.58\text{\%}$). The interaction term between cMFG thickness and age group was the sole significant predictor in this model (*β* = 1.67; *p* < 0.05). Simple slope analysis revealed a significantly greater relationship between cMFG thickness and associative memory performance in older compared with that in young adults (*β*_OA_ = 2.31, 95% CI [1.20, 3.42]; *β*_YA_ = 0.63, 95% CI [−0.34, 1.60]; Fig. 2*C*). The relationship between cMFG thickness and associative memory performance in older adults remained statistically significant after correcting for chronological age (*r* = 0.33; 95% CI [0.139, 0.514]; *p* < 0.005). No other linear models examining age differences in structure–behavior relationships reached statistical significance (*p*s > 0.004).

### Greater hippocampal and MFG structures relate to increased RSFC and enhanced source and associative memory in older adults

To investigate how age differences in brain structure and network connectivity interact to affect memory performance, we computed a B-PLS analysis. This analysis extracts latent patterns of connectivity related to both measures of performance (list recall, associative, and source memory) and brain structure (antHC, postHC, rMFG, and cMFG). This analysis revealed two significant LVs (*p* < 0.05).

**Figure 3.**
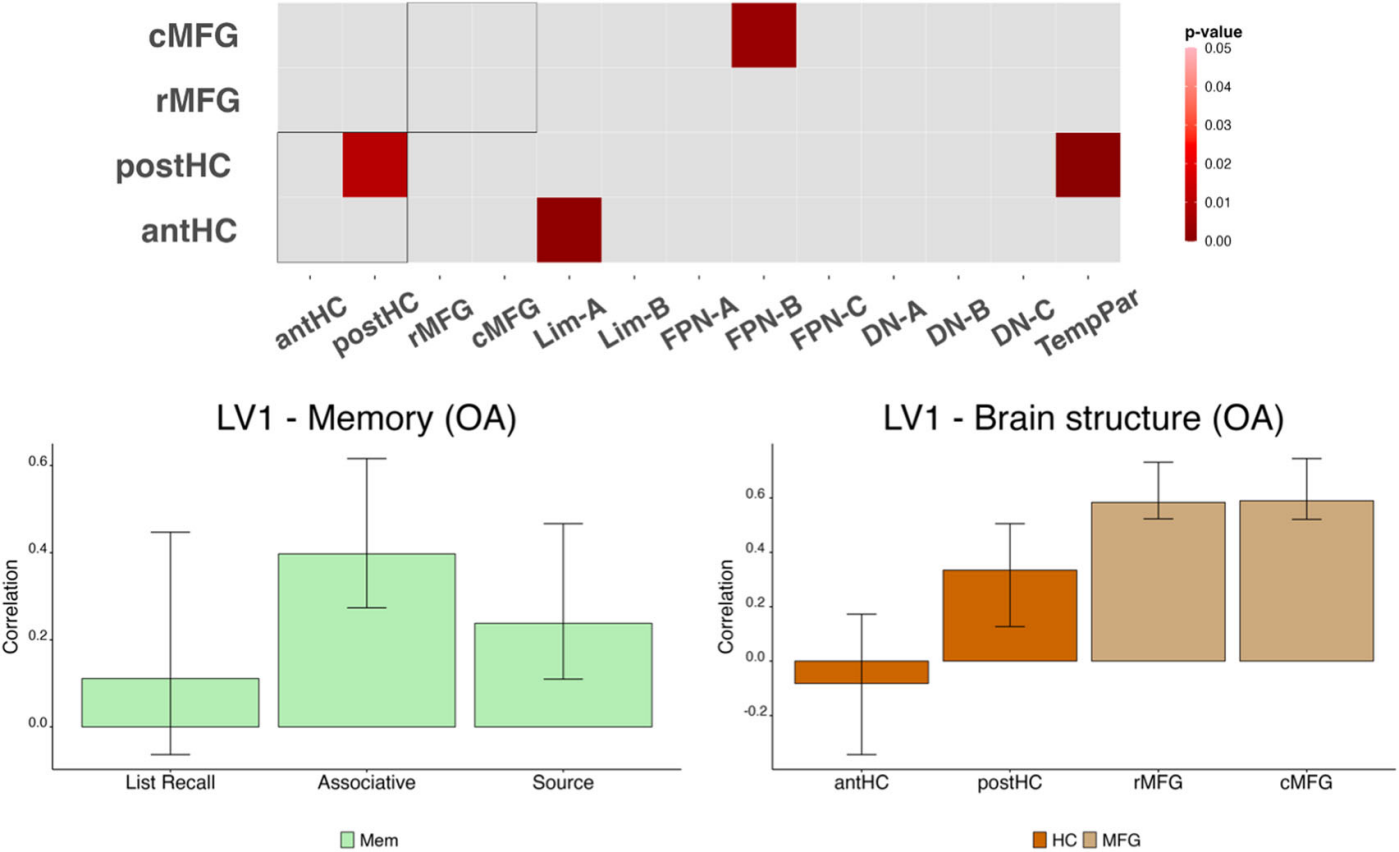
LV1: MFG thickness, postHC volume, and episodic memory performance relate to greater RSFC in older adults. B-PLS analysis revealed an association between memory performance, brain structure, and resting-state connectivity (25.69% cross-block covariance; *p* < 0.01). The connectivity summary plot display results of parcel-level analyses recapitulated at the network level. Memory performance and brain structure did not significantly relate to this pattern in young adults.

The first LV (LV1; Fig. 3) revealed a pattern of connectivity related to memory performance and brain structure that was specific to older adults (25.69% cross-block covariance; *p* < 0.01). Greater source and associative memory performance, as well as larger postHC and MFG structures, related to greater connectivity within the postHC and between the antHC and temporal pole (Lim-A). Enhanced ability on these two processes tapping relational memory was also related to greater connectivity between the postHC and the temporoparietal network, centered on semantic–linguistic regions across the temporoparietal junction (Catani et al., 2005; Tomasi and Volkow, 2012; Friederici and Gierhan, 2013). Greater connectivity between the cMFG and the FPN-B network was also related to better relational memory, as well as larger postHC and MFG structures. In particular, cMFG-FPN-B connectivity was subsumed by parcels within the left inferior parietal lobule (IPL) and left inferior temporal (IT) cortex within the FPN-B network. Brain scores from this LV were negatively correlated with chronological age in older adults (*r* = −0.26; *p* < 0.05). In other words, older adults with lower chronological age expressed this pattern of connectivity more than the older individuals in this age group.

### Smaller hippocampal volume and hypoconnectivity are related to better episodic memory in older adults

**Figure 4.**
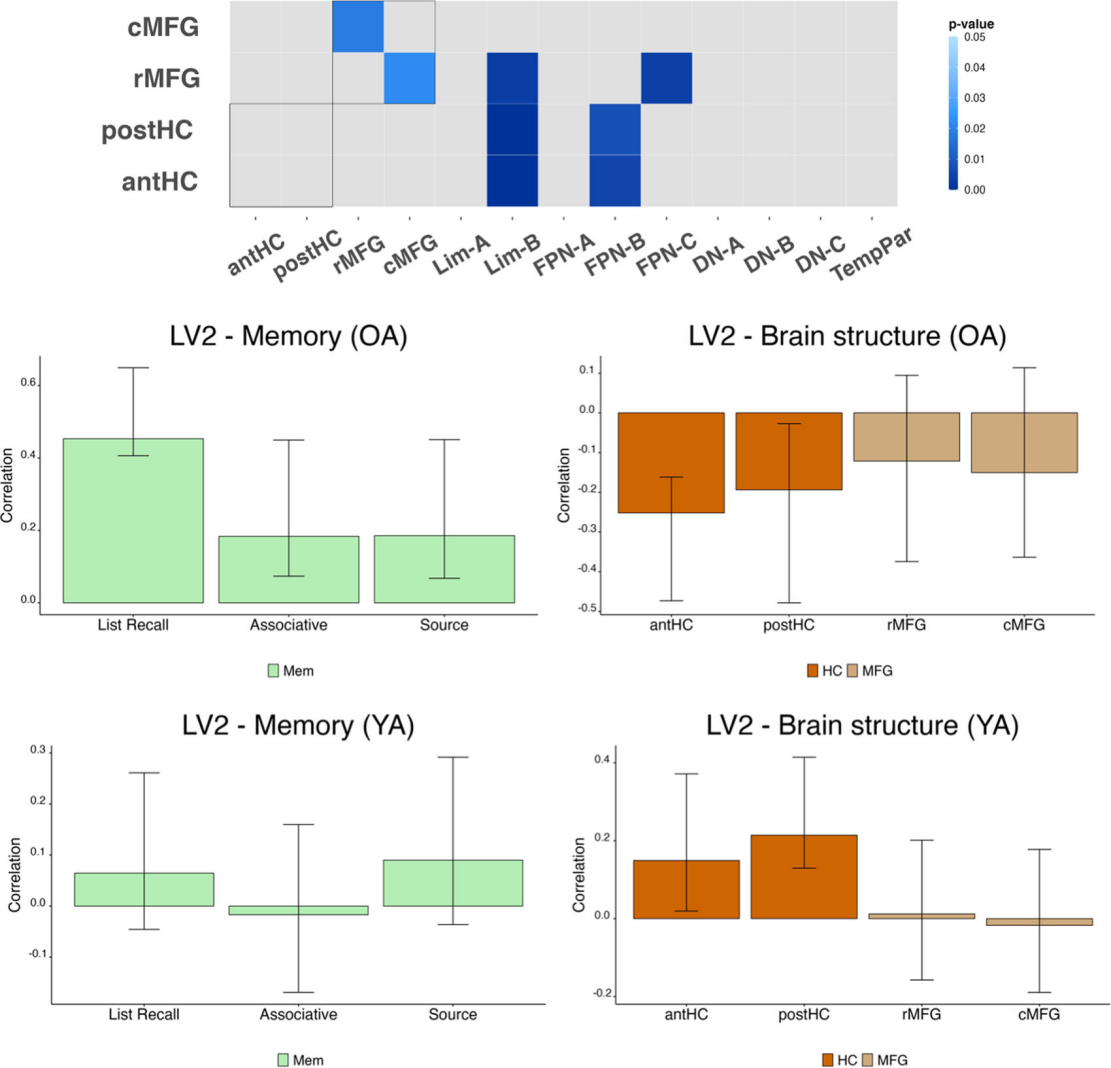
LV2: HC volume and all measures of episodic memory performance relate to pattern reduced RSFC in older adults. B-PLS analysis revealed an association between memory performance, brain structure, and resting-state connectivity (16.68% cross-block covariance; *p* < 0.05). All episodic memory processes and antHC and postHC volumes are related to this pattern in older adults; antHC and postHC volumes are inversely related to this pattern in young adults.

The second LV (LV2; Fig. 4) revealed an age group interaction. In older adults, decreased connectivity within this pattern of RSFC was associated with smaller antHC and postHC volumes and better performance across all episodic memory tasks (16.68% cross-block covariance; *p* < 0.05). This pattern included lower connectivity between the left rMFG and right cMFG and the right rMFG and left cMFG, lower connectivity between the left cMFG and right hippocampus, and between the bilateral OFC (Lim-B) and both the hippocampal subregions and rMFG. Lower connectivity between the hippocampus and the left IPL, left IT, and left medial PFC (mPFC) from the FPN-B network were also apparent. Finally, lower connectivity between the rMFG and the posterior cingulate cortex (PCC) and precuneus (PCun) parcels from the FPN-C network was related to reduced hippocampal volume and enhanced memory performance. This same RSFC pattern was associated with larger hippocampal volumes in younger adults, such that larger volumes were related to less connectivity at these same edges in this group, with no relationship to performance. Brain scores from LV2 were not significantly correlated with chronological age in older adults (*r* = −0.16; *p* = 0.17). This indicates that individual differences in hippocampal volume and functional connectivity, particularly reductions in both properties, can interact to contribute to greater memory performance in older adults, regardless of chronological age and regardless of the subtype of episodic memory.

## Discussion

The hippocampus and MFG are central to episodic memory function (Simons et al., 2005; Eichenbaum, 2017). As we age, the hippocampal and middle frontal subregions deteriorate (Raz and Rodrigue, 2006; Langnes et al., 2020), and large-scale functional networks become increasingly integrated (Chan et al., 2014). These well-studied neural signatures of aging parallel declines in episodic memory performance. While much previous work has examined these separate elements, the present study bridges the gap between these structural and functional properties to understand how distinct episodic memory processes are affected in the aging brain. First, we observed differential contributions of prefrontal structure to memory performance in younger and older adults. Next, we revealed patterns of connectivity related to episodic memory in older adults, tied to the structural integrity of the hippocampus and MFG. These interactions identified (1) how greater structural integrity and within-network functional connectivity relate to greater source and associative memory performance in older adults and (2) how functional reorganization characterized by a pruning of task-irrelevant connections, in the context of reduced hippocampal integrity, may be crucial to sustain memory performance in aging.

### Caudal MFG thickness is related to better associative memory performance in older adults

Aging comes with changes to distinct episodic memory processes, including memory for listed items, associations between items, and relationships between items and source information (e.g., trial type; Cansino, 2009). Aligned with our findings, the gray matter within the PFC has been shown to be especially crucial for associative memory in older adults, over and above medial temporal contributions (Becker et al., 2019; Brehmer et al., 2020; Guardia et al., 2023). This may be due to the role of this region in monitoring and linking multiple streams of information (Blumenfeld et al., 2011; Waltz et al., 1999). Indeed, the lateral prefrontal cortex is implicated in working memory and inhibition (Mitchell and Johnson, 2009; Owens et al., 2018), and the caudal MFG in particular has been shown to support top-down control of attentional selection (Germann and Petrides, 2020). Interestingly, the relationship we observed between cMFG thickness and associative memory was distinctive to older adults. The age specificity of this brain–behavior association may be related to the extensive changes across this region in late adulthood (Craik and Grady, 2002; Salat et al., 2004; Rajah and D’Esposito, 2005) as well as changes in inhibition, working memory, and attention that emerge in later stages of development, which may impact episodic memory (Miyake et al., 2000; Salthouse, 2019; Campbell et al., 2020; Ferguson et al., 2021).

While we did not observe associations between hippocampal volumes and memory performance in either sample, it is apparent that these structures support episodic memory across a variety of tasks (Palombo et al., 2018; Snytte et al., 2020). We may have observed a ceiling effect in young adults, both in terms of performance and in variability of structural data. A larger sample may be required to observe more of these subtle relationships in a group of individuals performing in a limited range (Marek et al., 2022). Still, the hippocampus is essential for storing memories of individual items and binding them with related information, which was observed when considering the intersection of structural and connectivity data.

### Greater associative and source memory performance relates to larger brain structure and within-network connectivity

Influential models of neurocognitive aging propose that decreases in neural resources may affect how the brain function supports memory performance (Reuter-Lorenz and Cappell, 2008). However, few studies have examined how functional organization relates to both memory performance and structural changes in key circuits (Fjell et al., 2016). In the present study, we observed a pattern of increased connectivity in older adults related to better source and associative memory performance and larger postHC and MFG structures.

We observed that greater connectivity within the postHC was related to greater structural integrity of the postHC and MFG and greater source and associative memory performance in older adults. The postHC has been functionally linked to both these types of memory in relating individual items to specific contexts (Nadel et al., 2013; Callaghan et al., 2021). We did not observe an effect of postHC volume on memory performance in older adults. However, we did find that greater postHC volume was linked with greater cross-hemispheric connectivity within this region. While some previous work indicates that greater hippocampal connectivity is linked to enhanced memory performance in older adults, this has not been consistent across studies, and assessing structure–function interactions may be critical to disentangle these differences (Wang et al., 2010; Sala-Llonch et al., 2014; Salami et al., 2014; Damoiseaux et al., 2016; Xu et al., 2019).

Greater connectivity between the antHC and temporal pole was also linked to better memory performance and larger postHC and MFG structure in older adults. Previous neurocognitive models of episodic memory have proposed a key relationship between the antHC and the anterior temporal lobe, crucial for linking semantic and schema-relevant information to episodic content (Nobre and McCarthy, 1995; Visser et al., 2010; Renoult et al., 2019). This functional alliance may be crucial for older adults, as aging comes with an increased reliance on conceptual processing to support episodic memory (Grilli and Sheldon, 2022). The structural integrity of the temporal pole has also been shown to support autobiographical episodic memory in older, but not younger, adults, indicating an underlying mechanism for the semanticization of cognition with aging (Spreng et al., 2018; Spreng and Turner, 2019; Setton et al., 2022a,b). Our results go further to reveal a potential component of functional organization (i.e., greater connectivity between the antHC and temporal pole) that may contribute to this process.

Enhanced connectivity of the cMFG with the FPN-B network was also related to this pattern of greater postHC and MFG structure and better source and associative memory performance. Given that the cMFG contains mostly parcels corresponding to the FPN-B network, greater expression of this pattern may indicate preserved within-network connectivity, and limited age-related network integration, a pattern typically associated with preserved cognitive functioning in older adults. Additionally, the frontoparietal control network is associated with executive control (Niendam et al., 2012). Greater connectivity within this network may contribute to memory performance by supporting executive functioning relevant for source and associative memory tasks.

All tasks examined in the present study contained relational demands (i.e., items were presented bound to a context, such as a list, another word, or a trial type) and displayed age-related deficits, consistent with past research (Spencer and Raz, 1995; Chalfonte and Johnson, 1996). The associative and source memory tasks that were correlated with this pattern of connectivity and structure tap this binding process to a greater degree than the list recall test, where contextual information is likely useful, but not required for performance (Sederberg et al., 2010). Critically, memory deficits in healthy aging are proposed to be linked with an impairment in associative binding (Old and Naveh-Benjamin, 2008). The functional configuration observed in this LV was more representative of a younger brain, as younger age was associated with greater expression of this pattern of connectivity in older adults. The link between this age-related pattern of connectivity and the associative and source memory tasks in particular suggests that this LV reveals a structure–function underpinning to the relational deficit that impacts memory performance in healthy aging. Another possible interpretation is that the chronologically younger individuals from the older adult sample, who displayed this enhanced young-like connectivity pattern, perform better on these two tasks because they provide a partial cue at retrieval. These scaffolds may be less beneficial for performance in the chronologically older individuals from the older adult group, due to their decreased expression of this connectivity pattern. Thus, expression of this functional assembly is associated with the ability of “younger” older adults to use partial cues to improve performance.

### Reduced connectivity is related to better performance in the context of smaller hippocampal volumes

A significant pattern of reduced RSFC related to better episodic memory performance in the presence of reduced hippocampal volumes (both antHC and postHC) emerged in older adults. We observed an asymmetrical pattern of connectivity across MFG subregions, such that lower connectivity between left rMFG and right cMFG and between the right rMFG and left cMFG was linked to better memory performance in older adults. Typically, older adults display greater bilateral activation in prefrontal regions during episodic memory tasks (Cabeza, 2002), though this is not always linked to better memory performance (Morcom and Henson, 2018). Here we observed that lower asymmetrical connectivity, perhaps reflecting less age-related differences in prefrontal function (Cabeza, 2002; Berlingeri et al., 2013), was related to better performance in older adults. Additionally, less connectivity between the hippocampus and FPN-B network was related to better performance. This result extends a key feature of neurocognitive aging (increased default-executive coupling in older adults) from cortical hubs to the hippocampus (Spreng and Turner, 2019). Furthermore, hippocampal volumes were inversely related to this pattern of connectivity in young adults. This interaction thus supports memory performance uniquely in older adults, suggesting that a potential functional reorganization of hippocampal and middle frontal connectivity may only benefit memory performance following structural changes in the hippocampus that present in late adulthood.

When previously essential neural resources become depleted, performance may be preserved via compensation by reorganization (Lövdén et al., 2010; Cabeza et al., 2018). This LV identified a common pattern of connectivity expressed in older adults who display better memory performance, in the context of reduced neural resources (smaller antHC and postHC volumes). Due to our correlational and cross-sectional methods, we cannot claim that this pattern of functional connectivity is playing a compensatory role as we could not observe whether changes in functional connectivity directly followed reductions in hippocampal volume. Still, this result provides insight into how functional reorganization may occur when hippocampal volumes are targeted in aging: as neural resources become sparse, it may become more efficient to reduce connections. Reduced connectivity could benefit cognition into two ways. First, if a reduction in connectivity occurs at specific edges but spares relevant alliances, it may be advantageous for memory performance. Indeed, connectivity within and between all edges shown to be related to enhanced relational episodic memory performance shown above are unaffected in this second pattern of reduced RSFC. Second, hypoconnectivity in the presence of enhanced cognitive functioning could be interpreted as the flip side of disease-related processes. Hyperconnectivity has been commonly observed early in age-related pathologies such as Alzheimer's disease (Schultz et al., 2017; Koelewijn et al., 2019; Bonanni et al., 2021) and is thought to be a fundamental response to neural disruption (Hillary et al., 2015). In examining a group of healthy older adults, the pattern of hypoconnectivity we identified may instead be protective against pathological aging. This may be particularly relevant for the OFC, which displays a broad pattern of hyperconnectivity in Alzheimer's disease (Zamboni et al., 2013). Here, we observed that lower OFC-hippocampal and OFC-rMFG connectivity were linked to better performance, suggesting that OFC hypoconnectivity may be a protective response to non-normative aging.

### Conclusions

In the present study, we report how age differences in the structural integrity and functional organization of the hippocampus and MFG interact and relate to memory performance. Much previous work has examined the contributions of age-related structural or functional brain differences to episodic memory independently. A vital step toward understanding the neural underpinnings of cognitive aging requires us to investigate how these fine-scale structural changes relate to larger-scale differences in functional connectivity. Previous work examining structure–function interactions has observed increased task-related frontoparietal activations in the presence of hippocampal structural declines (Maillet and Rajah, 2011; Snytte et al., 2022). The present study provides a novel angle in examining how aging impacts the interaction between the functional architecture of the default and frontoparietal control networks and the structural integrity of the brain regions essential for episodic memory (Fjell et al., 2016). By considering how these pieces move together, here we revealed neural signatures of aging that encompass two potential functional responses to structural differences with age, in relation to memory functioning. First, more gray matter in key structures and greater within-network connectivity together supported episodic memory in older adults. Second, in the presence of smaller hippocampal structure, we found that hypoconnectivity between regions and nonaffiliated networks was related to greater memory performance.

Observing how the healthy aging brain reorganizes to compensate for structural degeneration can provide insight into clinical disease progression. The pattern of hypoconnectivity we observed that supports memory processing diverges from the commonly hyperconnected brain that presents early in the disease course of major neurocognitive disorders. This provides a unique perspective on the complex and intersecting structural and functional trajectories that eventually converge to promote a shift from normative to non-normative aging. Understanding and mapping these trajectories will be essential to guide interventions aimed at delaying this transition.

## Synthesis

Reviewing Editor: Alexander Soutschek, Ludwig-Maximilians-Universitat Munchen Decisions are customarily a result of the Reviewing Editor and the peer reviewers coming together and discussing their recommendations until a consensus is reached. When revisions are invited, a fact-based synthesis statement explaining their decision and outlining what is needed to prepare a revision will be listed below. The following reviewer(s) agreed to reveal their identity: NONE. Note: If this manuscript was transferred from JNeurosci and a decision was made to accept the manuscript without peer review, a brief statement to this effect will instead be what is listed below.

Reviewer #1

The authors have couched their interpretation of the source memory measure appropriately, but this did not permeate their presentation in the abstract, where results are still described as "source memory" without qualification. As this is the only info many "quicker" readers will see, I think more cautious presentation in the abstract is still necessary. The authors have responded well to my other comments.

## Author Response

Response to Reviewer The authors have couched their interpretation of the source memory measure appropriately, but this did not permeate their presentation in the abstract, where results are still described as "source memory" without qualification. As this is the only info many "quicker" readers will see, I think more cautious presentation in the abstract is still necessary. The authors have responded well to my other comments.

We thank the reviewer for their comment. The reviewer correctly points out that we did not couch our description of source memory in the abstract. It has now been adjusted to indicate that correct performance on this task may be due to (1) correct recall of the word and its associated trial type, or (2) correct recall of the word in its presented sensory modality (visual or auditory). "In a sample of younger (N = 111, mean age = 22.11 years) and older (N = 78, mean age = 67.29 years) adults we analyzed structural MRI and multi-echo resting state fMRI data. Participants completed measures of list recall (free recall of words from a list), associative memory (cued recall of paired words) and source memory (cued recall of the trial type, or the sensory modality in which a word was presented)."
